# Clinical Usefulness of SISCOM-SPM Compared to Visual Analysis to Locate the Epileptogenic Zone

**DOI:** 10.3389/fneur.2020.00467

**Published:** 2020-05-29

**Authors:** Carla Oliveira Young, Elba C. S. C. Etchbehere, Edna Marina Souza, Sergio Querino Brunetto, Allan de Oliveira Santos, Mariana C. L. Lima, Sebastian Ortiz-De la Rosa, Marina Alvim, Clarissa Lin Yasuda, Celso Darío Ramos, Fernando Cendes, Barbara Juarez Amorim

**Affiliations:** ^1^Division of Nuclear Medicine, Department of Radiology, University of Campinas (UNICAMP), Campinas, Brazil; ^2^Center of Biomedical Engineering, University of Campinas (UNICAMP), Campinas, Brazil; ^3^Division of Epilepsy, Department of Neurology, University of Campinas (UNICAMP), Campinas, Brazil

**Keywords:** brain perfusion, SPECT, SISCOM, SPM, seizures, epilepsy

## Abstract

**Introduction:** Subtraction of ictal-interictal SPECT co-registered to MRI (SISCOM) is a quantification tool that can improve the sensitivity and specificity of the epileptogenic zone (EZ) localization. Commercially available image analysis software packages for SISCOM are costly, and Statistical Parametric Mapping (SPM) could be an alternative free software for the definition of the EZ. There are only a few studies that compare SISCOM using SPM (SISCOM-SPM) with visual analysis.

**Aim:** To compare SISCOM-SPM vs. visual analysis for localization of the EZ in patients with pharmacoresistant focal epilepsies.

**Materials and methods:** We evaluated all our patients with focal epilepsies that underwent ictal and interictal SPECT. We defined the reference standard to locate the EZ by pathology and follow-up (in patients submitted to surgery), or seizure semiology, serial EEG, long-term video-EEG, ^18^F-FDG PET/CT, and MRI (in patients who were not operated). We compared the location of the EZ by visual analysis of SPECT images and by SISCOM-SPM to the reference standard and classified as *concordant, discordant*, or *partially concordant*.

**Results:** We included 23 patients. Visual analysis was *concordant* with the EZ reference standard in only 13 patients (56.5%), while SISCOM-SPM was *concordant* in 18 cases (78.3%), providing a 21.8% increase in the location of EZ. However, this difference was not significant due to the small sample size (*p* = 0.0856).

**Conclusion:** Our preliminary results demonstrate that, in clinical practice, SISCOM-SPM has the potential to add information that might help localize the EZ compared to visual analysis. SISCOM-SPM has a lower cost than other commercially available SISCOM software packages, which is an advantage for developing countries. Studies with more patients are necessary to confirm our findings.

## Introduction

Epilepsy is a brain disorder characterized by an ongoing tendency for recurrent epileptic seizures (1). It is widely distributed, affecting between 0.5 and 1% of the world population (2). Approximately 30% of patients are refractory to medical treatment (2–4), and in these cases, surgical resection of the epileptogenic zone (EZ) is the treatment of choice (3). The determination of adequate surgical candidates, as well as the precise localization of the EZ, is complex and should be carried out by a specialized multidisciplinary team to obtain the best treatment response and minimize side effects (3–6).

Lesion localization requires high-resolution imaging and state-of-the-art image reconstruction software alongside neurophysiological, clinical, and seizure semiology assessments (7). Magnetic resonance imaging (MRI) is the most used imaging method to localize the epileptogenic lesion; however, it cannot determine the EZ in 20–30% of temporal lobe epilepsy and in 20–40% of extratemporal epilepsy (8). In this scenario, functional imaging methods play an essential role.

Brain perfusion using single photon emission computed tomography (brain SPECT) is a nuclear medicine functional neuroimaging method able to identify regional cerebral blood flow alterations caused by the EZ (9, 10). Brain SPECT can be performed during a seizure (ictal SPECT), showing increased perfusion in the EZ, and during the interictal period (interictal SPECT), showing decreased perfusion in the EZ (3, 5).

A meta-analysis has shown that the sensitivity for visual localization of temporal lobe EZ is 97% on ictal SPECT and 44% on interictal SPECT (11). For extratemporal lobe epilepsies, the sensitivity for ictal SPECT is ~66% (11, 12). However, sometimes visual analysis can be challenging (13).

Subtraction of ictal and interictal SPECT co-registered to MRI (SISCOM) has been proposed to increase the sensitivity and specificity in EZ detection (5, 6, 14, 15). SISCOM images have superior spatial specificity than ictal or interictal images individually and can adequately localize EZ even in patients with focal cortical dysplasia and a normal MRI (3, 16).

Unfortunately, commercially available image analysis software packages for SISCOM are costly. In contrast, the Statistical Parametric Mapping (SPM) is a free software that is well-documented in the literature to perform brain quantification (17–19).

However, there are only a few studies comparing SISCOM using SPM (SISCOM-SPM) vs. visual analysis (16, 19–21). The purpose of this study was to compare the ability to locate the EZ using SISCOM-SPM vs. visual analysis in patients with refractory epilepsy.

## Methods

This study was approved by the Ethics Committee of the Institution (CAAE: 87016717.0.0000.5404).

Patients referred from the epilepsy clinic to the nuclear medicine division between July 2015 and July 2018 were retrospectively reviewed. Inclusion criteria were patients with pharmacoresistant focal epilepsies according to the ILAE (International League Against Epilepsy) criteria (1), and who were submitted to ictal and interictal SPECT imaging. Exclusion criteria consisted of images with unsatisfactory technique.

We reviewed their medical data to determine seizure semiology, seizure frequency, serial electroencephalograph (EEG) recordings, long-term video-EEG monitoring, brain MRI, and positron emission tomography fused to computed tomography (PET/CT) with fluorodeoxyglucose labeled with fluorine-18 (^18^F-FDG).

### Reference Standard to Locate the Epileptogenic Zone (EZ)

The reference standard to define the location of the EZ was defined according to the following criteria:

- In patients who underwent surgery for epilepsy, we considered the epileptogenic lesion determined by an interdisciplinary investigation to be the surgical resection area. We provided the anatomopathological and postoperative follow up results in those who were operated in Table 1.
- In patients not submitted to surgery, the reference standard to define the location of the EZ was determined by a consensus during our weekly interdisciplinary patient management conference. Our interdisciplinary team has more than 20 years of experience and includes epileptologists, neuroradiologists with expertise in epilepsy, epilepsy-trained neurosurgeons, nuclear medicine physicians, and neuropsychologists. We discussed each case with all the patient's data and exams, including clinical history, seizures semiology (family description and the available videos), serial EEGs, long-term video-EEG monitoring, MRI findings, ^18^F-FDG PET/CT results.

**Table 1:** Data from all patients included in the study.

**Patient**		**Semiology**	**EEG**		**PET**	**MRI**	**AP**	**Follow up (Engel)**	**EZ (by Reference standard)**		**Injection time (s)**	**Visual analysis**	**SISCOM-SPM analysis**
**No**.	**Age range**		**Ictal**	**Interictal**					**Side**	**Lobe**		
1	31–35	Autonomic aura (dizziness), aphasia and impaired awareness	Left Fronto-Temp	Left Fronto-Temp	Left Temp-Par	Left Frontal CxThick	–	–	Left	Frontal	4	Discordant	Concordant
2	36–40	Right upper limb clonic movement, versive head posture to right side, impaired awareness	Left Fronto-TempRight FrontalGen	Bil FrontalGen	Right Temp	Right Fronto-Temp FCD	HS + FCD IIIA	III-A	Right	Temp	5	Concordant	Concordant
3	36–40	Jamais vù, bilateral manual automatisms	Left Temp	Left Temp Bil Temp	Left Temp	Left Frontal FCD	–	–	Left	Temp	5	Discordant	Concordant
4	21–25	Epigastric sensation aura, impaired awareness, right hand automatisms	Bil Fronto-Temp Right Fronto-Temp	Left TempBil Temp	Right Temp	Right Temp FCD	–	–	Right	Temp	10	Concordant	Concordant
5	26–30	Epigastric and right cephalic sensations, impaired awareness, bilateral manual automatisms, BTCS	Left TempRight Temp	Left Temp	Left Temp	Left Temp CxThick	–	–	Left	Temp	60	Concordant	Concordant
6	46–50	Verbal (repetitive speech) and bilateral manual automatisms, impaired awareness	Bil TempRight Temp	Bil Temp	Right Temp	Right HA	–	–	Right	Temp	3	Concordant	Concordant
7	46–50	Epigastric (malaise) and cephalic sensation (light headedness), impaired awareness	Left Temp	Left Temp	Bil Frontal^*^	EncBil Frontal^*^	–	–	Left	Temp	28	Concordant	Concordant
8	11–15	Atonic (trunk and head) seizures and BTCG	Right Temp-Par^*^	Right Temp-OcpLeft Temp-Ocp	Right Temp Right Ins	Right Ins FCD	–	–	Right	Ins	21	Discordant	Concordant
9	36–40	Epigastric sensation, impaired awareness, BTCS	Right Temp	Right Temp	NL	Right Temp FCD	–	–	Right	Temp	15	Concordant	Discordant
10	21–25	Epigastric sensation, aphasia, BTCS	Left Fronto-Par	Left Fronto-Temp	Left Temp-Ocp	Left Temp Enc	–	–	Left	Temp	4	Concordant	Concordant
11	1–5	Agitation (look for the mother), four limbs tonic posture	Right Frontal	Right HemRight Temp-Ocp	–	TS	Frontal, Ocp TS	I-A	Right	Frontal Ocp	10	Partially concordant	Concordant
12	26–30	Visual hallucination, impaired awareness, BTCS	Left Frontal	Left Fronto-Temp	Left Hem	Left Temp FCD^*^	–	–	Left	Frontal	22	Concordant	Concordant
13	31–35	Autonomic aura (thoracic discomfort), visual hallucination, clonic left upper limb and jaw movements	Right Hem	Right Fronto-Temp Right Par-Ocp	Right Hem > Ins	Right Hem Atrophy (>Temp + Amg)^*^	–	–	Right	Ins	13	Discordant	Concordant
14	26–30	Behavior arrest, BTCS (nocturnal seizures)	Left Frontal	Left Fronto-TempBil Frontal	Left Frontal	Left Frontal FCD	–	–	Left	Frontal	20	Discordant	Discordant
15	46–50	Autonomic aura (tachycardia, sweating), impaired awareness, BTCS	Right Temp	Right Temp	Right Temp	Right HA	–	–	Right	Temp	34	Concordant	Concordant
16	21–25	Right jaw tonic movement, right cephalic version, BTCS (nocturnal seizures)	Left Frontal	Bil Frontal	Left Temp-Ocp>Ins	Left Temp-OcpFCD^*^	–	–	Left	Ins	8	Discordant	Concordant
17	51–55	Emotional onset (fear), impaired awareness, BTCS	Left Temp	Bil Temp	–	Left HA	Left HS	I-A	Left	Temp	20	Concordant	Concordant
18	26–30	Behavior arrest, left ocular version, BTCS	Left Temp	Left Temp	Left Temp	Bil HA	–	–	Left	Temp	41	Concordant	Discordant
19	21–25	Atonic seizures/behavior arrest/Left upper limb clonic movements/facial clonic movements (majority left sided)/BTCS	Gen	MfGen	Bil Temp	Right Frontal FCD + Diffuse Atrophy	Callosotomy	III-A^*^	LeftRightRight	TempTempFrontal	14	Partially concordant	Partially concordant
20	31–35	Emotional onset (fear), autonomic sensation (thoracic discomfort), impaired awareness, BTCS	Bil Temp	Left Fronto-Temp	Bil Temp	NL	–	–	Left	Temp	–	Concordant	Concordant
21	16–20	Sensory (gustative) and Autonomic (sialorrhea) onset, bilateral clonic movements, impaired awareness, BCTS	Right Hem	Right Temp	Right Fronto-Temp	Right Frontal, Ins FCD	CE	III-A^*^	Right	FrontalTemp	28	Partially concordant	Discordant
22	21–25	Autonomic aura (vertigo), impaired awareness	Left Temp-Ocp	Left Temp	Left Temp Left Ins	Left Temp Post FCD	–	–	Left	Ins	2	Discordant	Concordant
23	51–55	Cognitive onset (jamais vù, aphasia), impaired awareness, BTCS	Left Temp	Bil Temp	–	Left HA	Left HS	I-A	Left	Temp	24	Concordant	Concordant

*Semiology description according to Blume et al. (22) and Fisher et al. (23)*.

*BTCS, bilateral tonic-clonic seizure; Bil, bilateral; Hem, hemisphere; Gen, generalized; Mf, multifocal; NL, normal; Temp, temporal lobe; Frontal, frontal lobe; Par, parietal lobe; Ocp, occipital lobe; Ins, insular lobe; Fronto-Temp, Frontal and temporal lobes; Post, posterior; Amg, amygdala; CxThick, focal cortical thickening; Enc, encephalomalacia; HA, hippocampal atrophy; HS, hippocampal sclerosis; FCD, focal cortical dysplasia; TS, Tuberous Sclerosis; CE, Chronic encephalitis; (>Temp + Amg), more evident in the Temporal lobe and Amygdala; (>Ins.), more evident in the Insula*.

* *cases explained in greater detail in the Supplementary Material*.

We classified the EZ as either temporal or extratemporal. We subdivided the extratemporal EZ into frontal, parietal, insular, and occipital regions. In patients with more than one suspected EZ, we considered their epilepsy as multifocal.

### Magnetic Resonance Imaging (MRI)

The MRI epilepsy protocol in a 3 Tesla Philips Intera Achieva scanner (Philips, Best, Netherlands) for visual analysis included:

Coronal images, perpendicular to the long axis of the hippocampus, defined at the sagittal image: (a) T2WI multi-echo image (3 mm thick, no gap, *voxel* size = 0.89 × 1 × 3 mm, TR = 3,300 ms, TE = 30/60/90/120/150 ms, matrix = 200 × 180, FOV = 180 × 180, TSE factor = 5; EPI factor = 5; flip angle = 90°; Geometry Corrected); (b) T1WI “inversion recovery” (3 mm thick, no gap, *voxel* size = 0.75 × 0.75 × 3 mm, TR = 3,550 ms, TE = 15 ms, inversion time = 400 ms, matrix = 240 × 229, FOV = 180 × 180, TSE factor = 7; Geometry Corrected), (c) Fluid attenuated inversion recovery (FLAIR) images [Fat-suppressed(FS) = SPAIR, FS Power = 1, 4 mm thick, slice gap = 1 mm, *voxel* size = 0.89 × 1.12.4 mm, TR = 12,000 ms, TE = 140 ms, inversion time = 2,850 ms, matrix = 180 × 440, FOV = 200 × 200, Geometry Corrected]; Axial images parallel to the long axis of the hippocampus: FLAIR images (FS = SPAIR, FS Power = 1, 4 mm thick, slice gap = 1 mm, *voxel* size = 0.89 × 1.12.4 mm, TR = 12,000 ms, TE = 140 ms, inversion time = 2,850 ms, matrix = 224 × 160, FOV = 200 × 200, Geometry Corrected); T1WI volume: with isotropic 1 mm *voxels* acquired in the sagittal plane (1 mm thick, no gap, flip angle = 8°, TR = 7.0 ms, TE = 3.2 ms, matrix = 240 × 240, FOV = 240 × 240, Geometry Corrected); T2WI volume: with isotropic *voxels* of 1.5 mm, acquired in the sagittal plane (no gap, TR = 1,800 ms, TE = 340 ms, matrix = 140 × 140, FOV = 230 × 230, TSE factor = 120; flip angle = 90°; Geometry Corrected).

Whenever MRI images were negative, additional sequences were acquired and analyzed:

T1WI volume: isotropic *voxels* of 1 mm, acquired in the sagittal plane (1 mm-thick, no gap, flip angle = 8°, TR = 7.0 ms, TE = 3.2 ms, matrix = 240 × 240, FOV = 240 × 240); FLAIR 3D, acquired in the sagittal plane (fat-suppressed = spectral-attenuated inversion recovery): FOV: 250 × 250, *voxel* size = 1.2 × 1.2 × 1.0 mm, TR = 4,800 ms, TE shortest, TI = 1,650 ms; T2WI 3D, acquired in the sagittal plane: no gap, FOV = 230 × 230, *voxel* size = 1.5 × 1.5 × 1.5 mm, TR = 1,800 ms, TE shortest; T1WI inversion recovery (3 mm-thick, no gap, *voxel* size = 0.75 × 0.75 × 3 mm, TR = 3,550 ms, TE = 15 ms, TI = 400 ms, matrix = 240 × 229, FOV = 180 × 180, TSE factor = 7); DIR 3D (double inversion recovery), acquired in the axial plane perpendicular to the long axis of the hippocampus: *voxel* size = 1.2 × 1.2 × 0.6 mm, TR = 5,500 ms, TE shortest, TI 2,550 ms; SWI 3D (Susceptibility weighted imaging) acquired in the axial plane perpendicular to the long axis of the hippocampus = *voxel* size = 1 × 1 × 1 mm, FOV 230 × 182, TR/TE = shortest.

The images used for co-registration with SPECT were the T1WI defined above.

### Interictal and Ictal SPECT

#### Patient Preparation for Interictal SPECT Imaging

All patients remained in a low-light, quiet room for 15 min, with a permanent intravenous access through a butterfly connected to a catheter with saline solution. While at rest, 1110 MBq (30 mCi) of ^99m^Tc-ECD were injected. The patients rested for another 10 min before the SPECT acquisition.

#### Patient Preparation for Ictal SPECT Imaging

All patients were monitored with a long-term video-EEG. The antiseizure medication was reduced in selected cases, to increase the chance of epileptic seizures. Patients rested while continuous video-EEG were recorded. All patients remained with permanent intravenous access through a butterfly connected to a catheter with a saline solution. To ensure a fast injection, a syringe with the radiotracer was connected to the catheter and protected with a lead shield. Upon seizure onset, around 1110 MBq (30 mCi) of ^99m^Tc-ECD were quickly injected. Seizures were confirmed by EEG and video recordings. SPECT images were acquired 30–90 min after cessation of seizure and patients' symptoms.

#### Brain SPECT Acquisition

SPECT was performed using a Symbia® T2 SPECT/CT system (Siemens, Erlangen, Bayern, Germany) equipped with a high resolution, low energy, two-head collimator, The SPECT images were acquired, with photopeak centered at 140 keV and 15% window, 128 × 128 matrix, 2.67 zoom (which could be variable), and 64 views for each head (37 s per view).

Raw data were reconstructed with 3D OSEM (17 intersections, 16 subsets) 7.65 mm Gaussian filter and CT attenuation correction. Images were displayed in transaxial, coronal, and sagittal planes for visual analysis.

#### Visual Analysis

Visual analysis was performed by two experienced nuclear medicine physicians with 20 and 23 years of experience in nuclear medicine brain images. These two nuclear medicine physicians evaluated the images in consensus looking for focal areas of hyper or hypoperfusion and comparing both cerebral hemispheres. They also compared brain perfusion with the cerebellar perfusion. Nuclear medicine physicians were aware of clinical and electroencephalographic findings.

The EZ was defined as a focal area of hyperperfusion in the ictal SPECT images, which became hypoperfused or normoperfused in interictal SPECT images. When there was more than one hyperperfused area, we correlated the findings with the time injection and ictal semiology to define the EZ. The other areas were considered as propagation areas.

### Subtraction Ictal-Interictal SPECT Co-Registered to MRI (SISCOM)

#### SISCOM Using SPM (SISCOM-SPM)

A trained physician performed the ictal and interictal subtractions using the Statistical Parametric Mapping (SPM) software, version 12 (SPM12) (Wellcome Department of Imaging Neuroscience, University College London, UK; available from http://www.fil.ion.ucl.ac.uk/spm/) in Matlab® (MathWorks, Natick, MA, USA) (24).

After the acquisition and reconstruction of interictal and ictal images, they were converted from DICOM to Analyze format using MRIcro software. Both ictal and interictal images in the Analyze format were loaded into SPM12, which runs in Matlab® software.

The images were subsequently realigned using the anterior commissure as reference. In the registration step, ictal, and interictal images were registered using routines based on Mutual Information Maximization algorithms. Both images were taken as target and source in two different registrations. The registered images were realigned, and the mean calculated, normalizing the uptake levels toward the whole brain. The positional correspondence of the registered images and their mean were checked in SPM12. After establishing that all areas were correspondent, the subtraction was done.

To obtain the cerebral perfusion differences, the transformed and normalized interictal SPECT image was subtracted *voxel*-by-*voxel* from the ictal SPECT image using the LCN12 subtract routine (24). The difference image was transformed into a *z*-score using the mean and standard deviation (SD) of the differences in all brain *voxels* (25).

The result of the subtraction was co-registered to the MRI image of the patient using the MRIcron software. All *voxels* exceeding two *z*-scores were considered significant. *Voxels* higher than two *z*-scores were considered hyperperfused. Usually, a hyperperfused region appears as a *cluster*, which is a set of *voxels*.

The EZ was considered as the *cluster* of hyperperfusion. When the *cluster* encompassed more than one lobe, we considered the *voxels* with the highest *z*-score as the EZ. When there was more than one hyperperfused *cluster* we correlated the findings with the time injection and clinical findings to define the EZ. The other areas were considered as propagation areas.

The subtraction operation took ~15 min.

#### Visual and SISCOM-SPM Analyses vs. Reference Standard

The location of the EZ obtained by visual and SISCOM-SPM analysis was compared to the reference standard and classified as:

- *concordant*, when there was an overlap between the hyperperfusion area and the EZ;
- *partially concordant*, when the analysis could not find all the EZ in patients with more than one EZ area; essentially, in patients with multifocal epilepsy;
- *discordant*, when no hyperperfused area was observed or there was an area of hyperperfusion in a different lobe from the EZ.

### Statistical Analysis

Descriptive analysis was performed for the numerical variables as age, gender, injection time, and percentage of concordance of visual and SISCOM-SPM analysis. Kappa coefficient and McNemar test was applied to evaluate agreement between visual and SISCOM-SPM analysis with the EZ reference standard. Mann–Whitney test was applied to compare the presence of seizure propagation foci with the radiotracer injection time. The level of significance adopted was 5%.

## Results

### Patients

Images of 24 patients were initially available for this study; however, one patient was excluded due to different ictal and interictal image acquisition parameters. Thus, 23 patients (12 women) with a mean age of 31.6 ± 12.9 years were included in the analysis (Table 1).

Among the 23 patients studied, 12 patients (52.2%) had extra-temporal lobe epilepsy, and 11 patients (47.8%) had temporal lobe epilepsy, according to the reference standard. Two patients had already been submitted to surgery before performing SPECT images. These patients had no seizures control and were under investigation for a new surgical approach.

After completing the investigation for EZ localization, including the SPECT images, six patients were submitted to epilepsy surgery. Sixteen (16) patients are still waiting for surgery, and one patient refused to operate. Histopathology of the cerebral specimens showed: focal cortical dysplasia (*n* = 2), hippocampal sclerosis (*n* = 2), tuberous sclerosis (*n* = 1), and encephalitis (*n* = 1).

The radiotracer mean injection time was 17.8 s ± 14.3 (range: 2–60 s).

### EZ Results

**Figure 1:**
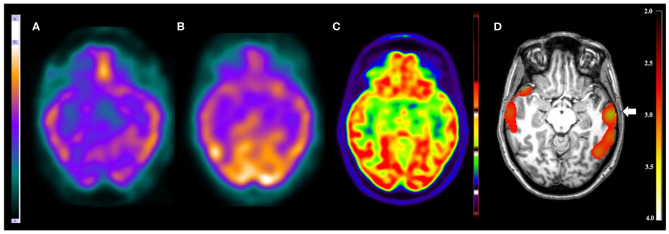
Patient #3 had pharmacoresistant epilepsy. The seizures began at 8 months of age, presenting with 1–2 seizures per week and in some periods 3–5 seizures per day. The EEGs showed epileptiform and non-epileptiform abnormalities in the left temporal region. MRI showed a mild thickening in the opercular region in the left frontal lobe. The reference standard determined the epileptogenic zone to involve the left frontal and left temporal lobes. **(A)** The ictal and **(B)** interictal SPECT images visual analysis were inconclusive. The **(C)** ^18^F-FDG PET/CT scan showed normal metabolism. However, the **(D)** SISCOM performed with SPM showed a *cluster* of hyperperfusion (arrow) in the left temporal lobe, which was compatible with the suspected regions by all studies and the reference standard.

**Figure 2:**
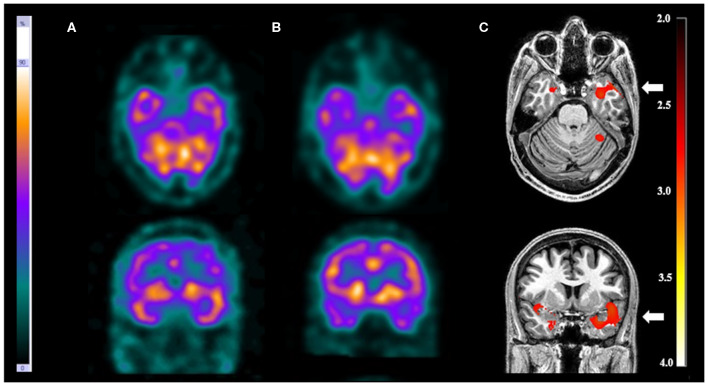
Patient #5 had pharmacoresistant epilepsy. Axial sections on first line and coronal sections on second line of **(A)** ictal SPECT and **(B)** interictal SPECT visual analysis showed a possible epileptogenic zone in the left temporal lobe. **(C)** SISCOM performed with SPM confirmed a focal area of hyperperfusion in the left temporal lobe (arrow). The reference standard suggested left temporal lobe epilepsy.

**Figure 3:**
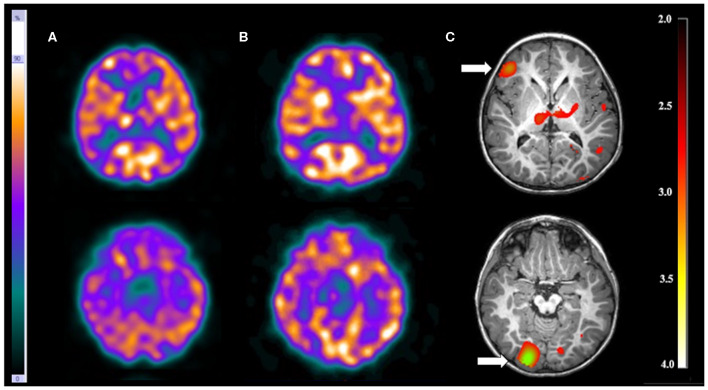
Patient #11 had tuberous sclerosis and refractory epilepsy. The axial images of the **(A)** ictal SPECT and the **(B)** interictal SPECT visual analysis showed a possible epileptogenic zone in the right frontal lobe. **(C)** The axial images in the SISCOM performed with SPM showed two foci of hyperperfusion in the right frontal and right occipital lobes (arrows). MRI showed the largest tubers in these two regions, and compatible with the main epileptogenic zone identified by EEGs and long-term video-EEG. This patient was submitted to surgery with resection of both tubers, resulting in cessation of seizure.

SISCOM-SPM was *concordant* with the reference standard in 18 cases (78.3%), while visual analysis was *concordant* in only 13 cases (56.5%). Therefore, SISCOM-SPM offered an increase of 21.8% in the EZ localization compared to visual analysis. However, this difference was not significant (*p* = 0.0856; Table 1). Figures 1, **3** show two cases that SISCOM was *concordant*, and visual analysis was *discordant* or *partially concordant*, and Figure 2 shows a case that both visual and SISCOM were *concordant* with the EZ.

SISCOM-SPM was *discordant* with the reference standard in four patients (17.4%) and *partially concordant* in one patient (4.3%). Visual analysis was *discordant* in seven patients (30.4%) and *partially concordant* in 3 (13.1%).

Moreover, younger patients showed significantly more *discordant* results (*p* = 0.0076) in visual analysis, while patients' age had no significant correlation with the SISCOM-SPM results.

There were three patients (13.0%) with more than one EZ. Visual analysis was able to detect just one of the EZ in each patient. On the other hand, SISCOM-SPM was able to detect all EZ in one patient (patient 11) who had tuberous sclerosis. In this patient, the visual analysis showed a suspicious EZ in the right frontal lobe while SISCOM-SPM identified two foci: one in the right frontal lobe and the other one in the right occipital lobe, compatible with the reference standard. This patient was submitted to surgery with resection of both tubers and became seizure-free (Figure 3).

### Seizure Propagation

Regarding visual analysis, no areas of EZ propagation occurred for injections performed below 15 s during the ictal study (*n* = 16; 69.6%). The presence of areas of EZ propagation was significantly (*p* = 0.0248) influenced by the radiotracer injection time. Areas of EZ propagation were present in seven patients (30.4%), all of them with injection times >15 s.

SISCOM-SPM analysis demonstrated areas of EZ propagation in 13 patients (56.5%). There was no influence of radiotracer injection time comparing with the presence of propagation areas (*p* = 0.3323).

## Discussion

Visual analysis of ictal and interictal SPECT can localize the EZ based on changes in cerebral blood flow (3). Ictal SPECT has a high sensitivity to detect the EZ for mesial temporal lobe epilepsy (above 90%) (11). However, in extratemporal epilepsies, the hyperperfused area in an ictal SPECT may be very mild and difficult to identify solely on the visual analysis (19, 21). In some patients, the hypoperfused area in the interictal SPECT can become normoperfused in the ictal SPECT (or less hypoperfused), without a clear area of hyperperfusion. In these patients, visual analysis can be quite challenging (6).

SISCOM can improve the sensitivity of visual analysis by showing a snapshot of cerebral hyperperfusion during a seizure compared to brain perfusion in an interictal period (26). The method is a non-invasive modality of imaging analysis that can detect the seizure focus with high spatial accuracy comparable to invasive EEG, but with significantly lower risk to patients (3). Previous studies have shown that SISCOM is valuable to localize the EZ in patients with extratemporal seizures and non-lesional MRI (14). Given the high incidence of extratemporal seizures in children, ictal SPECT could become particularly useful for surgical planning in this population (3).

Several studies have assessed the practical clinical value of SISCOM for preoperative evaluations, compared with MRI, PET, ictal EEG, EEG–functional MRI, and with the reference standard from surgical analysis (14). However, few papers in literature compare the SPECT visual analysis with SISCOM (16, 19–21). In addition, none have compared visual analysis to SISCOM-SPM. Some studies have compared SISCOM performed with the software Analyze™ and compared it with visual analysis. One study found a significantly higher rate of EZ localization with SISCOM compared to visual analysis (88.2 vs. 39,2%) (15). Similar results were found by Kaiboriboon et al. (21), in which SISCOM was concordant with EZ in 71.0 vs. 47.4% by visual analysis. In a meta-analysis (14), that evaluated the value of SISCOM in identifying EZ and predicting outcomes in epileptic patients that underwent surgery, in a total of 320 surgical patients, 275 (85.9%) had a positive SISCOM study. The authors concluded that SISCOM can provide complementary information, especially when MRI is negative. Although a few studies have not found the same good results for SISCOM, the number of patients included was small. There is a study, for example, analyzing 13 patients. Only eight patients were studied with SISCOM and 5 (62.5%) had the EZ located correctly. The visual analysis performed in all patients was consistent with the EZ in 11 of 13 patients (84.6%) (19).

The present study shows the initial experience using SISCOM-SPM in our epilepsy center. Our results using SISCOM-SPM agree with the studies that demonstrated an improvement in EZ detection using SISCOM (without SPM). In our study, SISCOM-SPM showed a higher sensitivity compared to visual analysis (78.3 vs. 56.5%, respectively) and provided a 21.8% increase in the EZ localization, which can be valuable in clinical practice.

We also found that younger patients showed more *discordant* results than older patients in visual analysis. This is probably because extratemporal lobe epilepsies are more frequent in children (27). In this group, seizures usually have a shorter duration and spread faster than temporal lobe epilepsy (28).

Regarding the practical aspects, the SISCOM-SPM imaging processing is an entirely automated method, totally reproducible, and compatible with the clinical practice. Moreover, the time to process the images (~15 min) was suitable for clinical routine, faster than other methods described in the literature (~30–45 min) with different softwares (21). Concerning the analysis of the SISCOM results, in cases with just one *cluster* of hyperperfusion, the analysis is straightforward. However, the analysis of cases with more than one hyperperfused area can be challenging. Sometimes, the larger *cluster* is not necessarily the EZ. Depending on the injection time, areas of propagation can be more prominent. Some studies already noticed that the SISCOM interpretation of different perfusion patterns is not straightforward (24) and they hypothesized that some regions of ictal hyperperfusion might represent a network of seizure onset and spread. A prerequisite for propagation within such networks is neuronal connectivity. This connectivity between seizure onset and ictal perfusion changes are not well-understood (26). In our study, when there was more than one hyperperfused *cluster*, we correlated the findings with the time injection and clinical findings to define the EZ in SISCOM. The other areas were considered as propagation areas. However, in many cases, SISCOM could not help, and some cases with multiple focal hyperperfused areas rendered the analysis inconclusive.

The present study has some limitations. First, the SPECT visual analysis was performed by two nuclear medicine physicians who were not blinded to the clinical context, which can introduce bias. However, this reflects a practical routine. The second limitation was the small number of patients included. The patient's workup in our epilepsy center includes a high-resolution MRI and interictal ^18^F-FDG PET/CT in addition to the clinical-EEG investigation. Only the cases in which these exams did not clearly detect the EZ were referred for an ictal SPECT. Moreover, this workflow also probably led to a bias in patient selection as ictal SPECTs were performed in more complicated cases. This bias also can explain the lower sensitivity of visual analyses in our study compared with the sensitivity from the literature. A third limitation was the small number of patients submitted to surgery (only six patients). Therefore, the EZ standard reference used in the majority of patients, the consensus during the interdisciplinary patient management conferences, is not considered a gold standard and can present some errors in EZ localization.

## Conclusion

In conclusion, our preliminary results demonstrate that, in clinical practice, SISCOM-SPM has the potential to add information that might help localize the EZ compared to visual analysis. It has a lower cost than other available SISCOM softwares, which is an advantage for developing countries. Studies with more patients are necessary to confirm our findings.

## Data Availability Statement

The raw data supporting the conclusions of this article will be made available by the authors, without undue reservation, to any qualified researcher.

## Ethics Statement

The studies involving human participants were reviewed and approved by Research Ethics Committee of the State University of Campinas (UNICAMP). Written informed consent from the participants' legal guardian/next of kin was not required to participate in this study in accordance with the national legislation and the institutional requirements.

## Author Contributions

Substantial contributions to the conception or design of the work, or the acquisition, analysis or interpretation of data for the work: CO, EE, and BA. Drafting the work or revising it critically for important intellectual content: CY, EE, ES, SB, SO-D, MA, FC, and BA. Agree to be accountable for all aspects of the work in ensuring that questions related to the accuracy or integrity of any part of the work are appropriately investigated and resolved: EE, MA, CY, SO-D, AS, ML, CR, FC, and BA. Provide approval for publication of the content: CO, EE, ES, SB, AS, ML, SO-D, MA, CY, CR, FC, and BA.

## Conflict of Interest

The authors declare that the research was conducted in the absence of any commercial or financial relationships that could be construed as a potential conflict of interest.
