# Supplementary material for: Clinical Usefulness of SISCOM-SPM Compared to Visual Analysis to Locate the Epileptogenic Zone
Source: Front Neurol. 2020 May 29;11:467. doi: 10.3389/fneur.2020.00467 (PMC7273921; doi:10.3389/fneur.2020.00467)
Supplement: Supplementary file 1 [file Data_Sheet_1.docx]

Supplementary Material

# Detailed explanation for some cases (Table 1):

Case #7: The changes presented in MRI and PET in bilateral frontal regions are due to areas of encephalomalacia. This region did not correlate with the semiology and EEGs findings, being not considered for EZ.

Case #12: This patient presented interictal EEGs with electrographic alterations on the left frontal lobe and left temporal lobe. Moreover, the long-term video-EEGs showed several seizures beginning in the left frontal lobe. The FDG PET/CT showed a more diffuse hypometabolism in the left-brain hemisphere. The MRI showed a mild alteration on the upper temporal gyrus suspected for focal cortical dysplasia. However, since PET did not show a focal hypometabolism on this region, MRI alterations were mild, and the long-term-video-EEG were very consistent with several seizures beginning in the left frontal lobe; we considered the left frontal lobe as the most probable EZ. This case is still being evaluated for surgical decision.

Case #13: This patient has reduced volume and signal in the caudate nucleus, hypersignal in the amygdala, and the adjacent portion of the hippocampus, associated with diffuse atrophy of the right cerebral hemisphere. Despite presenting diffuse structural changes, the patient semiology seizures presents clonic movements on the face, and a chest oppression sensation that may correspond to insular semiology. Since the insula is a deep lobe, it could justify more diffuse changes.

Case #16: In this case, MRI shows a possible focal cortical dysplasia type I in the left temporo-occipital region. Ictal EEG shows seizures starting at the left central frontal region. PET shows a similar finding to MRI with a hypometabolism in the temporo-occipital region, however, more intense in the insular region. Since the semiology presented with clonic movements on the face, compatible with insular onset, the left insula was suspect for the EZ

Case #19: This patient presented different types of seizures semiology: dropp attack, behaviour arrest, left clonic movement, bilateral tonic-clonic seizure. The long-term video-EEGs were generalized and multifocal, however several seizures began in the right temporal lobe, left temporal lobe or bilateral temporal lobe. Although MRI presented a diffuse cortical atrophy, the right frontal lobe presented with thickness in pre-frontal gyrus being suspected for focal cortical dysplasia. The PET images also showed hypometabolism on bilateral temporal lobes. Therefore, although a diffuse alteration could be suspected, we considered 3 regions as the main responsible for EZ: bilateral temporal lobes and right frontal lobe. This patient was submitted to a callosotomy and now does not have atonic seizure.

Case #21: One of the reasons why this patient is not seizure-free is because he got arrested a few months after the surgery and is not taking the medication correctly.

Case #22: This patient’s ictal and interictal EEGs showed alterations in the left temporal lobe. On long-term video-EEG, it is noticeable that the seizure’s electrographic changes started only 28 seconds after the beginning of the clinical seizure. The PET/CT performed prior to surgery showed reduced uptake in the left temporal lobe, encompassing the insular lobe. This patient had already undergone a temporal lobe resection in the past. The pathological analysis showed dysplasia, and this patient was not seizure-free after surgery. After re-analyzing the case, we considered a deeper focus based on the ictal EEGs with electrographic onset only after many seconds of the clinical onset. Therefore, it is probable that the left insula is part of the epileptogenic zone which was not completely resected during surgery.
